# High-Performance Flexible PLA/BTO-Based Pressure Sensor for Motion Monitoring and Human–Computer Interaction

**DOI:** 10.3390/bios14100508

**Published:** 2024-10-17

**Authors:** Xuguang Sui, Qingmiao Mu, Jia Li, Bo Zhao, Hongxi Gu, Han Yu, Juan Du, Lijun Ren, Dengwei Hu

**Affiliations:** 1 Engineering Research Center for Titanium Based Functional Materials and Devices in Universities of Shaanxi Province, Faculty of Chemistry and Chemical Engineering, Baoji University of Arts and Sciences, Baoji 721013, China; sxg18681950587@163.com (X.S.); 18909177718@163.com (Q.M.); 18840316432@163.com (J.L.); yuhan@bjwlxy.edu.cn (H.Y.); renlijun.wind@163.com (L.R.); dwhu@bjwlxy.edu.cn (D.H.); 2 Department of Advanced Materials Science, Faculty of Engineering and Design, Kagawa University, Takamatsu 761-0396, Japan; s24d161@kagawa-u.ac.jp

**Keywords:** piezoelectric pressure sensors, barium titanate tetragonal, polylactic acid, motion monitoring

## Abstract

Flexible electronics show wide application prospects in electronic skin, health monitoring, and human–machine interfacing. As an essential part of flexible electronics, flexible pressure sensors have become a compelling subject of academic research. There is an urgent need to develop piezoelectric sensors with high sensitivity and stability. In this work, the high flexibility of polylactic acid (PLA) film and the excellent ferroelectric properties and high dielectric constant of tetragonal barium titanate (BTO) led to their use as filling materials to fabricate flexible piezoelectric composite films by spinning coating. PLA is used to produce flexible binding substrates, and BTO is added to the composite to enhance its electrical output by improving its piezoelectric performance. The peak output voltage of the PLA/BTO tetragonal piezoelectric film is 22.57 V, and the maximum short-circuit current was 3041 nA. Durability tests showed that during 40,000 s of continuous operation, in the range of 15~120 kPa, the linear relationship between pressure and the film was excellent, the sensitivity for the output voltage is 0.176 V/kPa, and the output current is 27.77 nA/kPa. The piezoelectric pressure sensor (PPS) also enables accurate motion detection, and the extensive capabilities of the PENG highlight its potential in advancing motion sensing and human–computer interactions.

## 1. Introduction

Nowadays, flexible electronics are receiving great attention due to their excellent wearability, flexibility, and compatibility with various substrates [1,2,3], showing wide application prospects in electronic skin, health monitoring, and human–machine interfacing. As an essential part of flexible electronics, flexible electronic sensors have the advantages of being soft, offering convenience, and having a low cost [4,5,6]. Recently, the high sensitivity [7], exceptional flexibility [8], simple structure [9], and rapid response [10] characteristics of flexible pressure sensors have been a compelling subject focus of academic research.

In general, pressure sensors could convert mechanical signals into electrical signals [11,12]. They can be categorized as piezoelectric [13], piezoresistive [14], capacitive [15], and triboelectric types [16]. Compared to other types of pressure sensors, piezoelectric pressure sensors use the piezoelectric effect of piezoelectric materials to respond to external mechanical signals/pressure [17,18,19], and they can realize self-power supply, factors which have attracted significant research attention. They have the advantages of high accuracy, long life, and reliability, can detect signals through a non-contact method, and can be widely applied for industrial automation [20], environmental monitoring [21], medical devices [22], and intelligent monitoring devices [23]. Additionally, self-powered piezoelectric sensors are considered as an attractive approach for solving environmental issues and energy supplies. Energy conversion can be realized from environmental energy to sensors, as the output signals they possess are persistent [24,25]. Therefore, piezoelectric sensors can generate electrical signals without an external power supply, and cannot cause pollution or release wastes [26,27].

To meet the above application needs, piezoelectric sensors should have two critical features: high flexibility and high sensitivity. Piezoelectric polymers are easy to process, soft, and low in cost [28], and examples such as polyvinylidene difluoride (PVDF) [29], polylactic acid (PLA) [30], and poly (*L*-lactic acid) (PLLA) [31] are outstanding candidates for use in flexible piezoelectric sensors. Li et al. developed a piezoelectric bending sensor for airflow speed sensing by fabricating spring-like structured core–sheath piezoelectric fibers (C-PEFs) [32]. The C-PEFs were prepared by directly electrospinning poly(vinylidene fluoride) (PVDF). The piezoelectric bending sensor displayed potential applications in bending, sensing, and human sleep behavior monitoring. However, a poor piezoelectric property and low sensitivity seriously confine its wide usage. In order to address the problems of piezoelectric polymers, some studies added different inorganic piezoelectric materials to piezoelectric polymers, obtaining piezoelectric composites. A significant piezoelectric performance boost could be observed, and thermal stability and flexibility are also improved. Min et al. prepared a wearable piezoelectric blood-pressure sensor (WPBPS) by combining a poly(dimethylsiloxane) (PDMS) passivation layer [33], a medical-grade adhesive layer and Pb(Zr_0.52_Ti_0.48_)O_3_ (PZT), which exhibited high sensitivity, a fast response time, and outstanding mechanical stability. Nevertheless, at present, further studies and in-depth research on piezoelectric composites are still required, especially focusing on mechanisms, biocompatibility, versatility, and stability.

PLA is widely used in the medical field, and has the advantages of degradability, biocompatibility, and low density. On the other hand, polylactic acid is a kind of piezoelectric polymer widely used at present, which has good mechanical properties and processing properties. Barium titanate (BTO) is widely used in the manufacture of multi-layer ceramic capacitors, supercapacitors, thermistors, iron appliances, and piezoelectric devices due to its excellent dielectric, ferroelectric, and insulating properties.

In this study, PLA/BTO piezoelectric nanocomposite films are prepared by a simple rotating coating method using two crystal structures of BTO. By contrasting the two films, the output characteristics, relative dielectric constants, ferroelectric characteristics and piezoelectric coupling constants were analyzed. The mechanisms of the superior piezoelectric property of the films were discussed by the multi-physics coupling unit simulation. Further, the energy harvesting ability of the nanocomposite was evaluated, and the film served as a piezoelectric pressure sensor (PPS), and the application of the motion and health monitoring was investigated.

## 2. Materials and Methods

### 2.1. Materials

Polylactic acid (PLA) granules were procured from Suzhou Ren fu Plastic Co., Ltd., Suzhou, China, barium titanate (BTO) cubic (average diameter ≈ 1 μm). Tetragonal barium titanate (average diameter ≈ 600 nm) was supplied by Yu mu New Materials Co., Ltd., Ningbo, China. Methylene chloride (CH_2_Cl_2_) was sourced from Energy Chemical Technology Co., Ltd., Guiyang, China.

### 2.2. Preparation of PLA/BTO Piezoelectric Nanocomposite Films

The PLA solution (17.4 ωt%) was prepared by dissolving 4.6 g of PLA powders into 20 milliliters of CH_2_Cl_2_ under magnetic stirring and ultrasonic methods. Then, different amounts of BTO (0–10 ωt%) were added to the PLA solution and sonicated for 3 h at a temperature of 0–4 °C to ensure the even distribution of BTO in the PLA solution. To produce the PLA/BTO piezoelectric nanofilms, a rotating coating machine was utilized. The solution of PLA/BTO was spin-coated onto a circular glass substrate measuring (11 cm × 11 cm). Initially, the solution was spread evenly by rotating at a slow speed (500 r/min), and then at a higher speed (1500 r/min) to create a film of consistent thickness. The specimens were subsequently loaded into a vacuum drying chamber and subjected to annealing at a temperature of 60 °C and a pressure of 1.013 × 10^−5^ Pa for 12 h. Next, the samples were immersed in absolute ethanol for an additional 12 h.

### 2.3. Fabrication of PPS Based on PLA/BTO Membranes

During the device assembly process, the central layer was created using the PLA/BTO film, and copper fiber tape measuring 3 × 1.5 cm^2^ was used as the top and bottom electrodes on both sides of the film. Each electrode was then connected to two wires using conductive tape. To provide electrical insulation for the PPS, a 125 mm polyethylene terephthalate (PET) film was placed over the two-electrode setup. Finally, for improved mechanical durability, the entire generator was wrapped in polyimide (PI) tape.

## 3. Results

### 3.1. Material Characterization, Preparation, and Characterization of Thin Films

**Figure 1:**
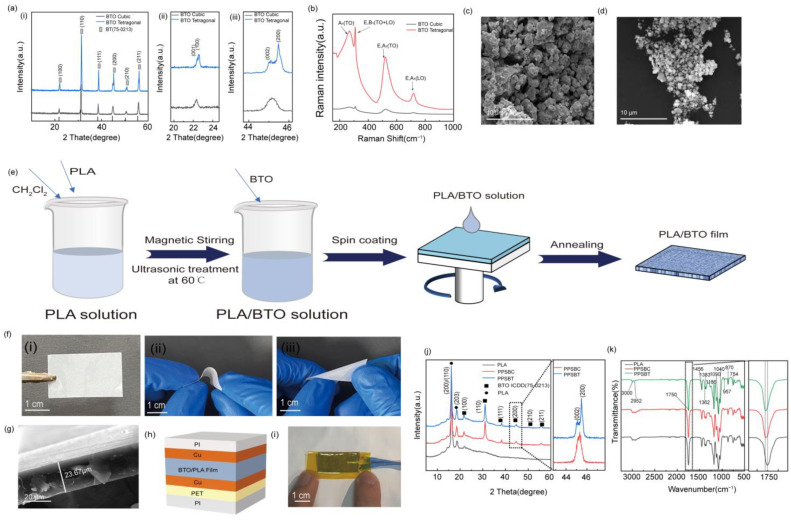
(**a**(**i**)) XRD spectra of BTO cubic and BTO tetragonal; (**a**(**ii**)) partial enlargement of the XRD spectrum at 22–24°; (**a**(**iii**)) 44–46° partial enlargement of the XRD spectrum; (**b**) Raman spectrum of BTO cubic and BTO tetragonal; corresponding SEM images of the (**c**) BTO cubic, (**d**) BTO tetragonal; (**e**) production process of piezoelectric composite film; (**f**) optical images of the flexible film; (**f**(**i**)) overhead optical images of both (**f**(**ii**,**iii**))flexure; (**g**) SEM image of film thickness; (**h**) structural diagram of PPS; (**i**) optical diagram of PPS; (**j**) XRD spectra of the samples; (**k**) infrared spectra of the samples.

As shown in Figure 1a, the crystalline phases of two kinds of BTO were analyzed, BTO cubic and BTO tetragonal. The strong and sharp diffraction peaks located at 22.15, 31.54, 38.94, 45.24, 50.87, and 56.13 for the two types of BTO are observed, which correspond to (100), (110), (111), (200), (210), and (211) crystal planes, and this agrees well with the crystal structure of the BTO material (ICDD No. 75-0213). Significantly, compared with BTO cubic, the splitting peaks of tetragonal BTO nanometers at 2θ = 22.16° and 45.25° are apparent for BTO tetragonal, as shown in Figure 1a(ii,iii) [34]. Raman spectroscopy was used to further determine the structure of the two samples. Figure 1b shows there are obvious optical differences between BTO cubic and BTO tetragonal. The Raman spectra of the two types of BTO are consistent with previous studies [35], showing the obvious peaks of longitudinal (LO) and transverse (TO) modes at 256 cm^−1^ [A_1_(TO)], 306 cm^−1^ [E, B_1_(TO+LO)], 513 cm^−1^ [E, A_1_(TO)], and 715 cm^−1^ [E, A_1_(LO)] [36,37]. It can be clearly seen that the Raman scattering intensity of BTO tetragonal is significantly higher than BTO cubic, which may be due to the particularity of its special crystalline structure. In addition, the piezoelectric performance of the BTO is strongly related to crystal structures. The morphologies of BTO cubic and BTO tetragonal were characterized by scanning electron microscopy (SEM), as shown in Figure 1c,d. The BTO cubic appears to have a globular morphology, and a level of aggregation occurs lead to the particle size are not uniform (0.8~1 μm). The particle size of the BTO tetragonal is more uniform than that of the BTO cubic, which is about 500~600 nm.

To investigate the energy harvesting performance of different barium titanates, the PLA/BTO piezoelectric nanocomposite films was fabricated by spin coating. As shown in Figure 1e, PLA, BTO, and CH_2_Cl_2_ were fully mixed during preparation, and the nanocomposite films were produced by the rapid rotation of the spinning machine. Subsequently, an annealing process is carried out to remove excess organic components. Finally, the PLA/BTO piezoelectric nanocomposite films were obtained. The digital photograph in Figure 1f shows the nanocomposite film is white in color, like barium titanate powder. The flexibility of the nanocomposite film is superb; it can withstand bending in all directions and shows high deformability (Figure 1f(ii,iii)). The nanocomposite film can withstand severe and frequent impact stress when used as a pressure sensor. Figure 1g shows the SEM cross-section image of the nanocomposite film. The thickness of the film is about 23.67 μm. Figure 1h shows the schematic diagram of the PPS construction based on the PLA/BTO piezoelectric nanocomposite film and the optical image of PPS is shown in Figure 1(i). The PLA/BTO nanocomposite film are used as the intermediate layer, the two sides of the device are wrapped with copper conductive tape. Notably, the upper and lower two layers of copper conductive tape should not contact to prevent the upper and lower electrodes of the device from short-circuiting. A PET film is attached to one side of the conductive tape, and a classic sandwich structure is formed to enhance the mechanical properties of the device, which helps the device to better accept different stress hits. Finally, the whole device is completely sealed with PI tape to prevent dust and moisture in the air from polluting and damaging the device.

The PLA/BTO piezoelectric nanocomposite films were studied by X-ray diffraction (XRD). The PPS with BTO cubic and BTO tetragonal are abbreviated as PPSBC and PPSBT, respectively. Figure 1j shows the two diffraction peaks in the range of 17–19° were assigned to the (200)/(110) and (203) crystal planes of PLA [38]. In addition, distinct diffraction peaks corresponding to BTO crystal faces (100), (110), (111), (200), (210), and (211) were also observed. The split peaks at 44–46° could be assigned to the BTO tetragonal, which indicates the PPSBT is successfully fabricated. While the PPSBC only could be found only one peak, which is consistent with the previous analysis. Fourier infrared spectroscopy (FT-IR) was used to further study the phase composition of the nanocomposite films. (Figure 1k) The asymmetric stretching vibration of the methyl-C-H bond was detected to be 2952 cm^−1^, and the symmetric stretching vibration to be detected was 3000 cm^−1^. The main polarity of polylactic acid can be attributed to C=O, and 1750 cm^−1^ can be attributed to its stretching vibration. The absorption peak of methyl asymmetrical bending vibration was 1456 cm^−1^, and the symmetric bending vibration absorption peak was 1383 cm^−1^. The symmetric tensile vibration of C-O-C in polylactic acid was observed at 1186 cm^−1^, and the absorption peak of asymmetric tensile vibration was 1090 cm^−1^. The tensile vibration absorption peak of the C=O group shifts to the long wavelength direction when BTO is added to PLA, which can be clearly observed from the enlarged view of Figure 1k. This may be due to the polymer chain carbonyl group (-C=O···O=C-) and BTO. The methyl absorption peak strength of the nanocomposites increased with the addition of BTO fillers, which may be due to the interaction between polylactic acid methyl and inorganic particles, resulting in the change in dipole moment [39,40,41]. The surface morphology of the nanocomposite films was characterized by scanning electron microscopy. Figure S1 shows the film was relatively flat after adding BTO fillers, with no obvious bulge or depression. As shown in Figure S2, the element mapping of PLA/BTO nanocomposite films reveals that with the addition of BTO fillers, barium and titanium elements become more obvious, showing a uniform distribution of these elements.

### 3.2. Electrical Properties of the PLA/BTO Nanocomposite Films

**Figure 2:**
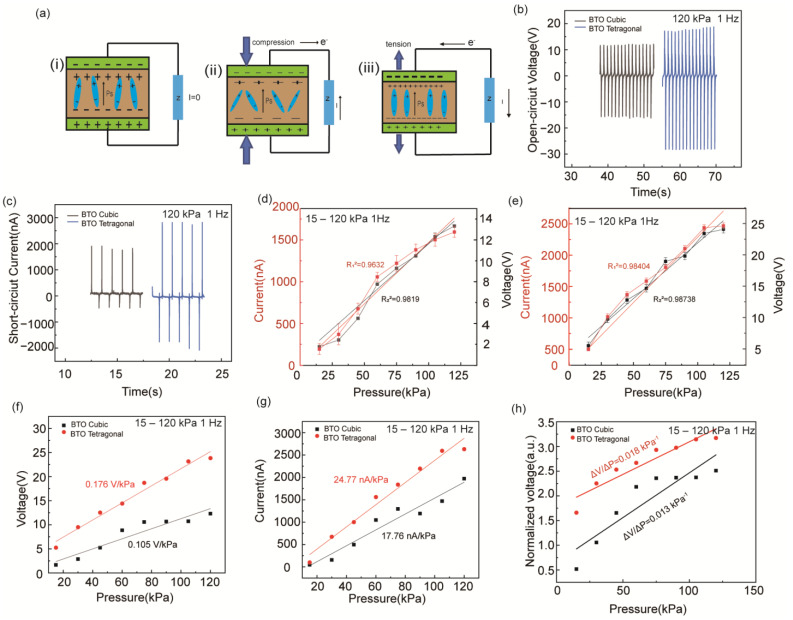
(**a**) Schematic diagram of the working principle of flexible PPS; (**b**) comparison of VOC of PPS prepared by using two different films; (**c**) comparison of ISC of PPS prepared by using two different films; impact stress and electrical output linear relationship diagram of capacity of (**d**) PPSBC and (**e**) PPSBT; (**f**) open-circuit voltage increment comparison between the two films; (**g**) comparison of short-circuit current increments of the two films; (**h**) linear comparison of the pressure and electrical output capacity of the two films.

Figure 2a illustrates the principle of the piezoelectric effect. The PPS mainly comprises three films that generate a positive or negative charge on their surface when deformed by a force in a specific direction. These charges are then transferred to the system via conductive tape and wires, the output performance of piezoelectric nanocomposite films is evaluated (Figure 2a(i)). When the sample is subjected to stress, a charge is generated by electrostatic induction, resulting in a piezoelectric potential (Figure 2a(ii,iii)). The piezoelectric output can be examined and analyzed using an electrometer. The setup of the piezoelectric test system is shown in Figure S3, illustrating the process of extrusion and release. The electrical output performance of the PENG was evaluated through a cyclic extrusion and release process. According to Figure 2b,c, when a cyclic force (120 kPa, 1 Hz) is applied to the PPS, the open-circuit voltage and short-circuit current are generated. The electrical output performance of PPS prepared with the BTO tetragonal exceeds that of the BTO cubic system. The BTO deforms under external mechanical stress, causing the misalignment of the centers of positive and negative charges, thereby generating piezoelectric potential [42]. Figure 2d,e show the correlation between impact forces and the growth of open-circuit voltage and short-circuit current of the PPSBC and PPSBT. The linear relationship between impact stress and electrical signal of the PPSBC is a little poor, and the R_1_^2^ and R_2_^2^ values are 0.9632 and 0.9819, respectively. The PPSBT has a more intuitive linear relationship between shock stress and electrical signal, with R_1_^2^ and R_2_^2^ values of 0.9840 and 0.9874. This shows that the PPSBT can better control the open-circuit voltage and short-circuit current. The strong linear relationship between the PPSBT and stress has more potential in a variety of sensing applications. As shown in Figure 2f–h, the sensitivity and sensitivity of the PPS under different pressure conditions is revealed. The sensitivity of the PPSBC is 0.105 V/kPa and 17.76 nA/kPa, respectively, which is significantly lower than the PPSBT (0.176 V/kPa and 24.77 nA/kPa, respectively). It could be attributed to the ability of BTO tetragonal to produce more electrical energy under the same pressure conditions. After performing the calculations, the PPSBT demonstrates excellent sensitivity up to 0.018 kPa^−1^, compared with the PPSBC (0.013 kPa^−1^).

### 3.3. The Mechanism of Electrical Signal Output Enhancement

**Figure 3:**
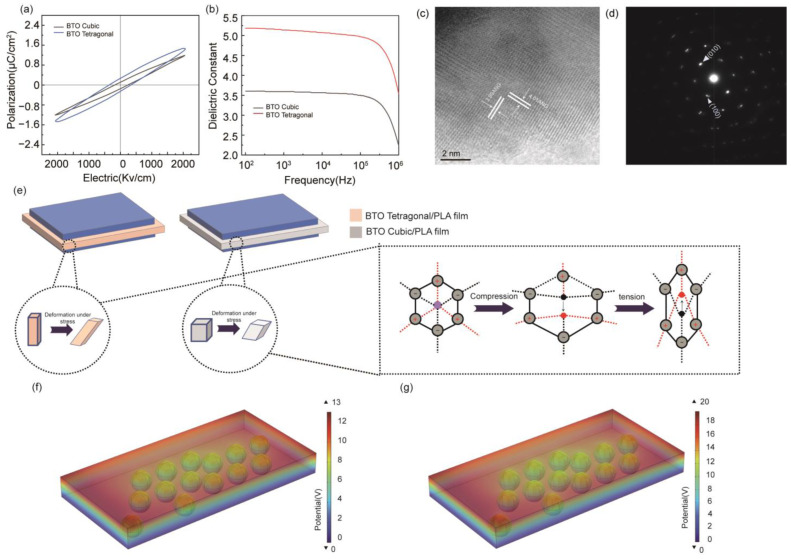
(**a**) Comparison of ferroelectric properties of the two films; (**b**) Comparison of Relative dielectric constant of the two films (**c**) HRTEM image of BTO tetragonal; (**d**) SAED image corresponding to BTO tetragonal; (**e**) mechanism diagram of BTO tetragonal enhanced piezoelectric output; (**f**) COMSOL simulation diagram of PPSBC; (**g**) COMSOL simulation diagram of PPSBT.

The hysteresis loops of the PLA/BTO nanocomposite films at 2000 Kv/cm^2^ field strength were measured in Figure 3a. The residual polarization of the PPSBC is 0.411, while that of the PPSBT is only 0.289, which indicates BTO tetragonal has better ferroelectric properties. As shown in Figure 3b, the relative permittivity (ε_r_) of PPS was analyzed from 0.1 kHz to 1 MHz at room temperature. When the frequency is increased, the dielectric constant of the PPS decreases, which is described as the Maxwell–Wagner–Sillar polarization effect [43]. The energy harvesting performance of piezoelectric materials (d_33_, piezoelectric coupling coefficient) is related to its relative dielectric constant and residual polarization, which is calculated as follows [44,45]:

d_33_ = 2Q_11_ ε_r_ε_0_P_r_ (1)

The piezoelectric coupling constant d_33_ of different filler materials is calculated by Formula (1). It is found that the addition of BTO tetragonal can effectively improve the ferroelectric and piezoelectric properties of nanocomposite films.

The microstructure of BTO tetragonal was further analyzed by transmission electron microscopy. According to Figure 3c, the lattice spacing is 3.99 Å and 0.401 Å, which correspond to the (100) and (001) planes. Figure 3d shows that BTO tetragonal is defect-free, and the normal direction is along [001], as observed in the selected area electron diffraction. Subtle differences in lattice parameters demonstrate the tetragonal symmetry of the material [46].

Piezoelectric effects can be divided into two types: positive piezoelectric effects and inverse piezoelectric effects. The positive piezoelectric effect refers to the deformation of piezoelectric crystal under the action of external force, resulting in the relative displacement between the positive and negative charge centers inside the crystal, resulting in polarization. In this process, an equivalent bound charge of the opposite sign will appear in a specific direction of the crystal, and its charge density is proportional to the external force applied. The basic mechanism of positive piezoelectric effects is shown in Figure 3e. Compared with BTO cubic, BTO tetragonal shows a higher aspect ratio, which makes the relative displacement between the positive and negative charge centers inside its crystal larger, resulting in a more significant polarization effect, generating more bound charges, and further improving the electrical output capacity of the PPSBT.

To investigate the piezoelectric response mechanism of PLA/BTO cubic and PLA/BTO tetragonal piezoelectric composite films, the stress distribution and piezoelectric potential were analyzed by COMSOL 6.1. Two PLA/BTO nanocomposite films were constructed by the multi-physics module of the piezoelectric device in COMSOL, and the piezoelectric distribution of the piezoelectric composite was simulated (Figure 3f,g). The BTO is evenly distributed in the PLA substrate. It can be seen from the electric potential distribution diagram under the same stress impact that the piezoelectric potential (20 V) generated by BTO tetragonal as the filler is significantly higher than that generated by BTO cubic (13 V). Figure S4i,ii show the stress distribution of films prepared with different fillers. While being hit by the same stress, the stress distribution of the film made of BTO cubic is less uniform than that made of BTO tetragonal, and the local stress generated is also lower. This may be due to the larger aspect ratio of BTO tetragonal compared to BTO cubic. When subjected to the same stress, BTO tetragonal can enhance the stress transfer, and the enhancement of surface stress can effectively enhance the piezoelectric response.

### 3.4. Electrical Properties of the PLA/BTO Tetragonal Nanocomposite Films

**Figure 4:**
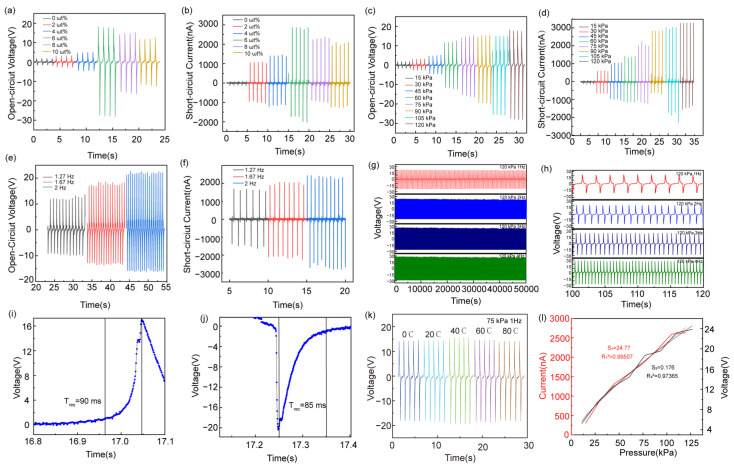
(**a**) VOC and (**b**) ISC of PPSBT at BTO concentrations of 0, 2, 4, 6, 8, and 10 ωt%; (**c**) VOC and (**d**) ISC of PPSBT (BT content of 6 ωt%) under different pressures; (**e**) VOCs and (**f**) ISCs driven by different frequencies; (**g**) durability tests of PPSBT under different operating frequencies (0~5000 s); (**h**) enlarged view of partial 100–140 s area data in (**g**); (**i**) response time of PPSBT under 75 Kpa; (**j**) recovery time of PPSBT under 75 Kpa; (**k**) power output capacity of PPSBT at different temperatures under 75 Kpa; (**l**) sensitivity of PPSBT.

The electrical output performance of the PPSBT was further optimized with the addition of different amounts of BTO tetragonal. According to Figure 4a,b, the content (0 ωt%–10 ωt%) of BTO tetragonal significantly impact the properties of the PPSBT. When the content of BTO tetragonal increases from 0 ωt% to 10 ωt%, the open-circuit voltage and the short-circuit current tends to increase first and then decrease, and the maxima are 20.17 V and 2021 nA at 6 ωt% BTO tetragonal. When the content of BTO tetragonal increases, the performance enhancement of the PPSBT may be due to the higher dielectric constant of the nanocomposite. Moreover, the addition of BTO tetragonal may enhance the dipole motion of polylactic acid and improve the overall piezoelectric properties. However, the open-circuit voltage and short-circuit current of the PENG tend to decrease when using more than 6 ωt% of BTO tetragonal, which may be due to the leakage phenomenon of the composite film [47,48,49]. More BTO tetragonal can initiate aggregation, and increases the rigidity of the film. The dipole motion of PLA under the same pressure is limited, leading to weakening the polarization of the PPSBT [50,51]. Therefore, the content of BTO tetragonal is chosen 6 ωt% in the PPSBT for further piezoelectric sensing application.

The piezoelectric sensing performance of the PPSBT was assessed (see Figure 4c,d). The open-circuit and short-circuit current increase as the cyclic impact stress increases from 15 kPa to 120 kPa at 1 Hz, which indicates excellent piezoelectric sensing performance. To study the influence of different strain conditions on piezoelectric sensing property for the PPSBT, different force conditions were applied. The frequency of reciprocating force was set to 1.27 Hz, 1.67 Hz, and 2 Hz, respectively.

As shown in Figure 4e,f, with the stress frequency increasing, the open-circuit voltage and short-circuit current also exhibit an increasing trend. The increase in electrical output capacity may be mainly due to the increase in induced charge generated by piezoelectric materials per unit time when the strain frequency increases, as in a piezoelectric capacitor, the higher the frequency, the less the discharge of the capacitor. Therefore, the higher the accumulated charge, the better the output performance. In addition, the piezoelectric signal is also small at low frequencies due to the mismatch between the impedance of the piezoelectric plate and the measuring system [52]. Continuous stress tests were performed to evaluate the mechanical durability of the PPSBT in Figure 4g,h. Under continuous stress, the electrical output performance of the PPSBT retains excellent stability without significant decline. The electrical output capacity does not show a significant decreasing trend at an open-circuit voltage of 26 V (see Figure 4g,h). These results show that the PPSBT has strong fatigue resistance and durability, which is suitable for long-term applications. As shown in Figure 4i,j, the response and recovery times of the PPSBT are 90 ms and 85 ms under 75 kPa, respectively. Additionally, as shown in Figure 4k, the electrical output performance of the PPSBT does not show significant changes under different temperatures, which indicates that the PPSBT shows excellent stability towards the temperature variation. Figure 4l shows the linear correlation curves between the electrical output and applying the pressure. In the range of 15~120 kPa, the output voltage and current increase, the sensitivity for the output voltage is 0.176 V/kPa, and the output current is 27.77 nA/kPa. From the above results, it can be seen that the sensitivity of the PPSBT is sufficiently high for pressure sensing. Furthermore, the performance of the PPSBT is comparable with results reported in the literature [53,54,55,56,57,58,59,60,61,62,63], details are shown in Table S1. According to Table S1, the PPSBT shows high sensitivity. Due to the high flexibility, the PPSBT displays a fast response and recovery time, good linear relationship between pressure and electrical output capacity, and a wide pressure monitoring range.

### 3.5. Applications of PPSBT

**Figure 5:**
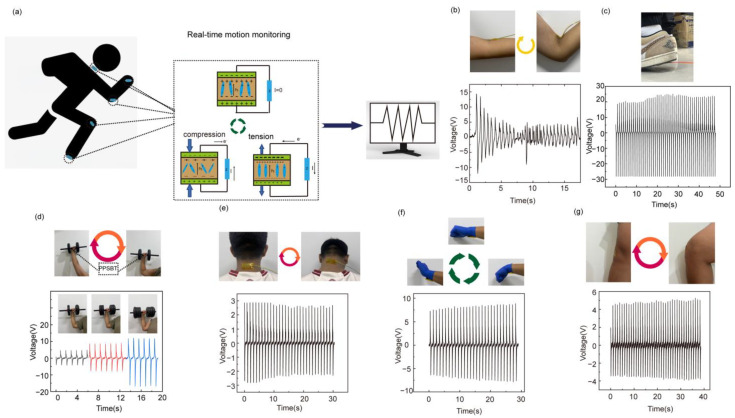
(**a**) Motion monitoring decoration diagram. Place PPSBT (**b**) at elbow; (**c**) on soles of the feet; (**d**) for lifting objects; (**e**) on the neck; and (**f**) on the wrist. (**g**) Optical images and electrical data of the knee joint.

Finally, the PPSBT was used for real-time motion monitoring, as shown in Figure 5. In this study, the PPSBT is installed on multiple parts of the human body, as shown in Figure 5a. When the human body is in a state of motion, the PPSBT installed on the human body will also generate mechanical deformation along with the movement of the human body. The PPSBT can effectively convert mechanical energy into electrical signals, and then realize the monitoring of human movement through computer analysis. We can see from Figure 5b that when the continuous motion process happens, the electrical signal shows obvious fluctuations; meanwhile, the voltage output also changes. In particular, when the PPSBT is attached to the sole of the foot, a stable power output of up to 20 V is observed under walking movement, as shown in Figure 5c. In addition, the PENG can also be used to monitor the pushing of dumbbells of different weights (Figure 5d). When the weight of the dumbbells increases, the electrical output increases correspondingly. Further, the PPSBT was attached to the neck, wrist, and knee joint. Joint motion can be clearly monitored through the electrical signals generated by the movement.

**Figure 6:**
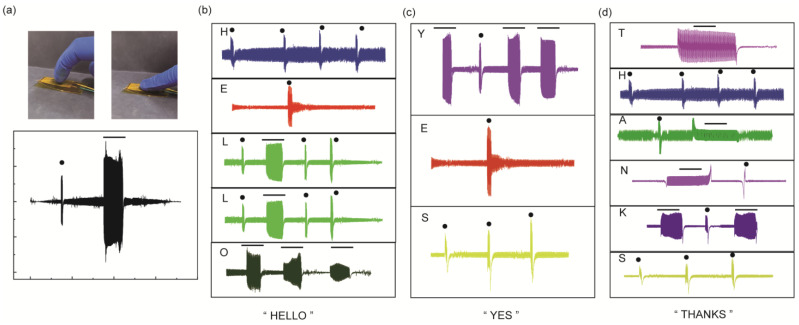
(**a**) Electrical signal response of PPSBT after different pressing time; (**b**) demonstrates the production of the Morse code “HELLO”; (**c**) demonstrates the generation of the Morse code “YES”; (**d**) demonstrates the generation of Morse code “THANKS”.

In addition, the application of flexible sensors in human–computer interaction systems is further studied. The PPSBT can generate a voltage signal when subjected to external force, and its output signal can remain relatively stable under the applied force state. As shown in Figure 6a, when the different force modes are applied, the force time of the PPSBT is controlled, and voltage signals of various shapes can be generated, such as “points” and “lines”. In standard International Morse code, dots and dashes in various permutations represent different letters of the alphabet. From this point of view, different sensing signals generated by different stress times can also be encoded accordingly. For example, as shown in Figure 6a, a short time of force will produce a voltage signal corresponding to the “point”, while a longer time of force will produce a voltage signal corresponding to the “line”. Therefore, by controlling the time of finger pressing, sensor signals with different waveforms can be generated, which represent the points and dashes in Morse code. The voltage signal output by the sensor can be convert into Morse code for information encryption and transmission. By rhythmic flexing and pausing of finger joints, the English words “HELLO”, “YES”, and “THANKS” are delivered, as shown in Figure 6b–d. The intelligent application of the PPSBT provides a new perspective for information transmission and intelligent interaction in the future.

## 4. Conclusions

In conclusion, two kinds of PLA/BTO piezoelectric nanocomposite films were fabricated by the spin coating technology, and PPSs were prepared. Compared to previous related research, the PPSBT exhibited outstanding piezoelectric performance, and the mechanism was analyzed by COMSOL 6.1. Subsequently, the optimal content of BTO tetragonal was 6 wt% for the PPSBT. When the stress was applied at 2 Hz and 120 kPa, the PPSBT exhibited an optimal output performance of 22.57 V and 3014 nA. In the range of 15~120 kPa, the output voltage and current increased, the sensitivity for the output voltage was 0.176 V/kPa, and the output current was 27.77 nA/kPa. In addition, after a 5000 s cycle of anti-fatigue testing, the film showed a stable output, proving its long-term reliability. Finally, the PPSBT was used for real-time motion monitoring, and different motion states could be detected. The PPSBT is also expected to find a wide range of applications in human–computer interaction.

## Data Availability

Data underlying the results presented in this paper are not publicly available at this time but may be obtained from the authors upon reasonable request.

## Supplementary Materials

The following supporting information can be downloaded at: https://www.mdpi.com/article/10.3390/bios14100508/s1, Characterization and Measurement; Figure S1: Electron microscope image of thin film surface; Figure S2: Element mapping image; Figure S3: Optical image of self-built piezoelectric detection device; Figure S4: COMSOL simulation of stress distribution in thin films; Table S1: Comparison chart of previous performance reports on pressure sensors.
