# Supplementary material for: High-Performance Flexible PLA/BTO-Based Pressure Sensor for Motion Monitoring and Human–Computer Interaction
Source: Biosensors (Basel). 2024 Oct 17;14(10):508. doi: 10.3390/bios14100508 (PMC11506190; doi:10.3390/bios14100508)
Supplement: Supplementary file 1 [file biosensors-14-00508-s001.zip › biosensors-3041639-supplementary.pdf]

biosensors

Supporting information

High-performance flexible PLA/BTO-Based Pressure Sensor for Motion Monitoring and human–computer interaction

Xuguang Sui [1], Qingmiao Mu [1], Jia Li [1], Bo Zhao [2], Hongxi Gu [1,*], Han Yu [1], Juan Du [1,*], Lijun Ren [1] and Dengwei Hu [1]

1 Engineering Research Center for Titanium Based Functional Materials and Devices in Universities of Shaanxi Province, Faculty of Chemistry and Chemical Engineering, Baoji University of Arts and Sciences, Baoji 721013, Shaanxi, China.

2 Department of Advanced Materials Science, Faculty of Engineering and Design, Kagawa University, Takamatsu 761–0396, Japan

* Correspondence: hello1207@163.com (H.G.); bwldj2010@163.com (J.D). Tel.: (optional; include country code; if there are multiple corresponding authors, add author initials)

Characterization and Measurement

Surface morphological structures of barium titanate (BTO) nanoparticles and piezoelectric nanofilms composed of PLA/BTO were examined using a scanning electron microscope (SEM, Quanta250 FEG, America) and a Raman spectrometer (inVia, England). The SEM images and energy-dispersive spectroscopy (EDS) elemental mapping were analyzed with an X-ray spectrometer (Genesis APEX APOLLO X, America). The micromorphology and interplanar spacing of the samples were analyzed using field emission transmission electron microscopy (TEM, JEM-2100) and high-resolution transmission electron microscopy (HRTEM, JEM-2100) from JEOL Company and FEI Company in the United States. A structural analysis of the atomic array on the sample was conducted using selected area electron diffraction (SAED). The chemical composition of the piezoelectric nanofilms was investigated using a Fourier transform infrared spectrometer (PerkinElmer, PE Spectrum Two, Germany) in the wavelength range of 4000 to 500 cm^$^{-1}$. The crystalline structures of BTO nanoparticles were determined through high-performance X-ray diffraction (XRD, DMAX U1TIMA IV, Japan) using Cu Kα radiation in the 5-90° range. The ferroelectric properties of the PLA-based film were tested using the American Premier II testing system. The result obtained was the P-E hysteresis loop of the film, which determined the residual polarization (Pr) of the film sample in μC/cm^2. The Pr value is essential for calculating the d33 (piezoelectric coefficient) of the film.

The open-circuit voltage and short-circuit current of the piezoelectric nanogenerator (PPS) were measured using a digital electrometer system (6514, Keithley, USA) and a computer in real time. The piezoelectric test system utilized in this study was a custom-made setup. This system involved a power drive that controlled a robotic arm with an impact head to apply a controllable force. The impact head could oscillate at a consistent frequency by adjusting the power drive. The force applied by the impact head onto the piezoelectric nanogenerator (PPS) was directed towards the sample. Additionally, a pressure sensor was fixed vertically on the sample plate by a stationary component to continuously monitor the pressure exerted by the impact head on the PPS. Due to the repetitive force from the impact head, electrostatic induction produced an electrical charge, resulting in the formation of an electric potential. Subsequently, the output performance of the piezoelectric device was evaluated using an electrometer on this reciprocating extrusion/release apparatus. The contact surface area measured 4.5 cm^2.

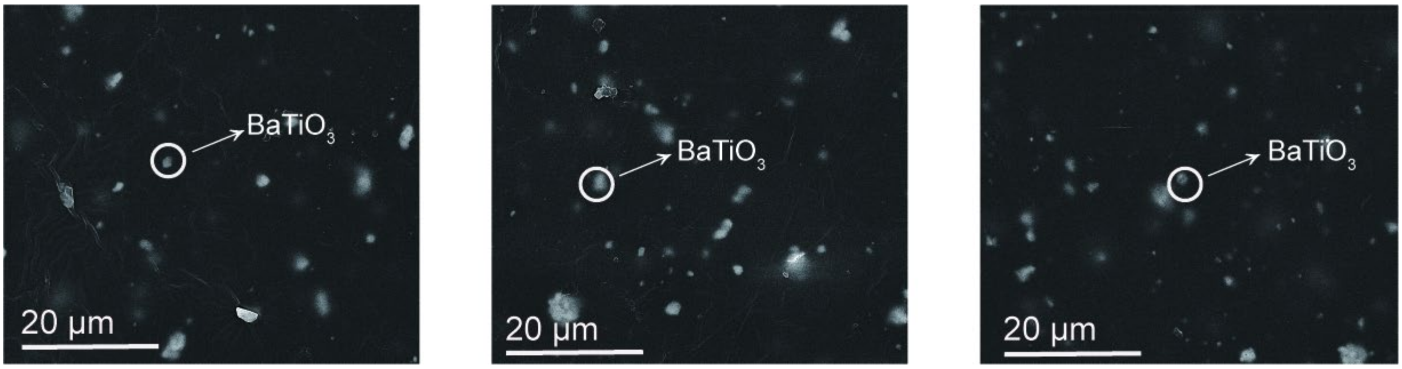

Figure S1. (a), (b), and (c) are SEM images of composite material planes under different views.

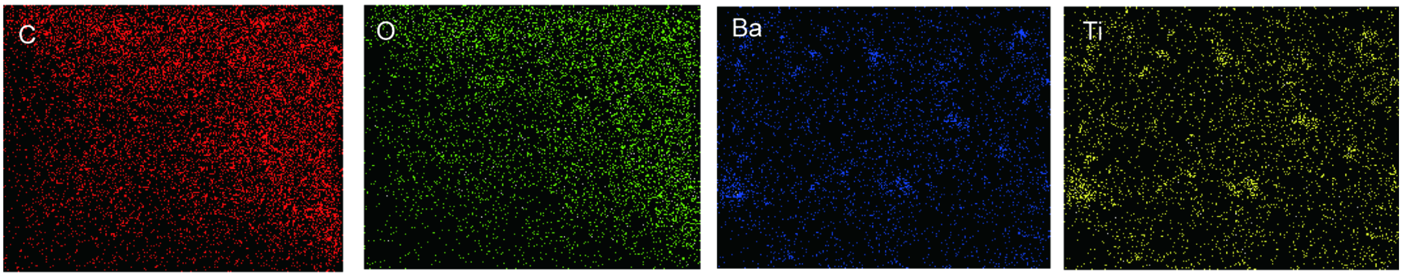

Figure S2. Element mapping for the SEM micrograph of composite materials

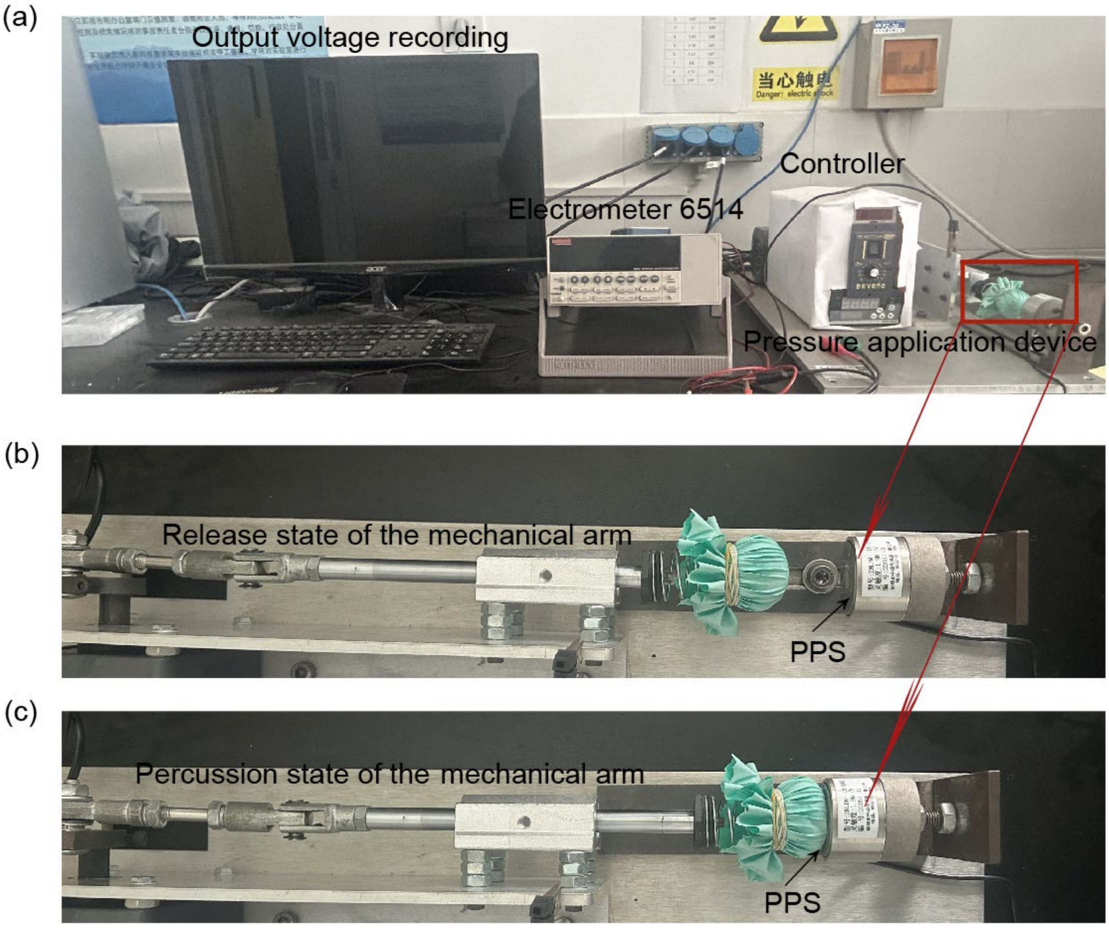

Figure S3. (a) Optical image of the self-built piezoelectric device test system. (b) The mechanical arm in its release state. (c) The mechanical arm in its percussion state.

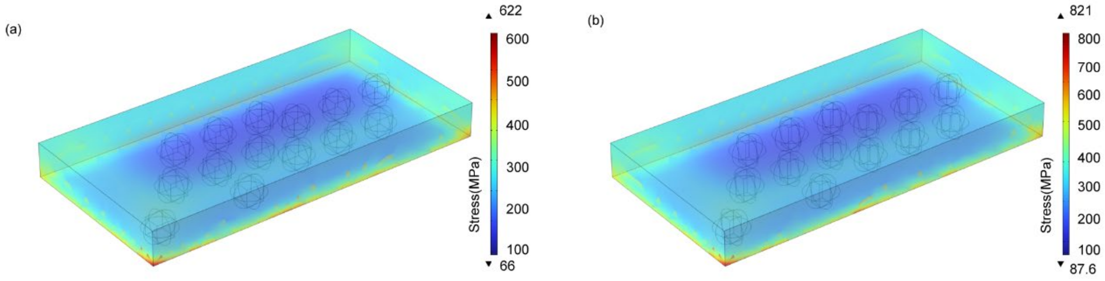

Figure S4. Stress calculation of COMSOL physical model. (a) Cubic barium; (b) Tetrag.

Table S1. Comparison chart of previous performance reports on pressure sensors.

Sensor type	Electrode material	Sensitivity	Monitoring range	Response time	Ref.
Piezoresistive sensor	MXene / PEDOT: PSS	754.5 kPa^{-1}<br>177.3kPa^{-1}<br>22.3 kPa^{-1}	0 - 0.8kPa<br>0.8 - 3.6kPa<br>3.6 -10.4 kPa	180/110 ms	[53]
Piezoelectric sensor	PDMS / ZnO	59.3 mV/kPa	0 - 3.5 kPa	—	[54]
Capacitive strain sensor	Copper	2 MHz / 1% ε	Strain 15%	—	[55]
Piezoelectric sensor	PVDF/BTO-NPs/AlN	5.713 V/N	0 – 1 N	4 ~ 6ms	[56]
piezoresistive sensors	CNFs/SBS/Ag NPs	769.2 kPa^{-1}<br>16.7 kPa^{-1}	0 – 2 kPa<br>2 – 18 kPa	100 ms	[57]
piezoresistive sensors	MXene/silver nanowires	1434.89 kPa^{-1}<br>285.03 kPa^{-1}<br>150.66 kPa^{-1}	0 – 5 kPa<br>5 - 25 kPa<br>25 - 100.5 kPa	70/81 ms	[58]
Piezoresistive sensor	PEDOT: PSS	—	Strain 150%	—	[59]
Piezoelectric sensor	MXene/Ag nanoparticle	2.2 Mv/0.1% ε	Strain 0.7%	—	[60]
Piezoresistive sensors	GNPs/MWCNT/PEO	2.69 - 14.05 kPa	0-70 kPa	100 ms	[61]
Piezoelectric sensor	Tellurium nanowire	25 mV/10% ε	Strain 50%	—	[62]
Capacitive strain sensor	OHP/ PEDOT: PSS/Si	1.0 kPa^{-1}	0 – 10 kPa	—	[63]
Piezoelectric sensor	PLA/BTO-T	0.176 V/kPa	0 – 120 kPa	90/85 ms	This work
